# Did the Medicaid expansion improve immunization among U.S. pregnant women?

**DOI:** 10.1016/j.pmedr.2025.103214

**Published:** 2025-08-20

**Authors:** Junying Zhao, Rashmi Jaggad, Ahmed El Fatmaoui, Pallab K. Ghosh

**Affiliations:** a Department of Health Administration and Policy, University of Oklahoma Health Sciences, 801 NE 13^th^ Street CHB 359, Oklahoma City, OK 73104, United States; b Department of Biostatistics and Epidemiology, University of Oklahoma Health Sciences, 801 NE 13^th^ Street, Oklahoma City, OK 73104, United States; c Department of Economics, University of Oklahoma, 308 Cate Center Dr, Norman, OK 73019, United States

**Keywords:** Affordable care act (ACA), Immunization, Medicaid expansion, Parity, Pregnant women, Vaccination

## Abstract

**Objectives:**

Affordable Care Act (ACA) Medicaid expansion increased financial eligibility of low-income individuals. Its effects are mixed on influenza vaccination and unknown for Tetanus, Diphtheria, and Acellular Pertussis (Tdap) vaccination among pregnant women in the United States. We aim to address mixed and missing evidence in literature.

**Methods:**

We merged 44,320 pregnant women from 2011 to 15 Pregnancy Risk Assessment Monitoring System with National Welfare Data by residency. Treatment group included Medicaid enrollees in post-ACA expansion states. Control included non-Medicaid enrollees in expansion states and all individuals in non-expansion states. We employed triple-difference method to estimate Medicaid expansion treatment effects on individual vaccination probabilities, and associations with socioeconomics and demographics.

**Results:**

Treatment group did not differ from control to vaccinate due to Medicaid expansion. Additionally, post-ACA Medicaid enrollees were 4.5–9 % less likely to vaccinate than pre-ACA non-Medicaid enrollees. Medicaid enrollees in expansion states were 2.5 % less likely to vaccinate against influenza than non-Medicaid enrollees in non-expansion states. African Americans were 4–5 % less likely, while Native Americans were 3–7 % more likely, to vaccinate than counterparts. Those with low parity and college education were 3.2–5.4 % and 4.8–10.6 % more likely to vaccinate. Individuals in high-poverty states were 0.6 % less likely to receive the influenza vaccine.

**Conclusions:**

We contributed different national data on understudied populations and the triple-difference method to the literature. Income-based Medicaid expansion did not increase the likelihood of vaccination among pregnant women. Non-income-based policies may target Medicaid-enrolled pregnant women in expansion states for influenza vaccination. Future interventions may target high-parity, low-education, and African American pregnant women in high-poverty non-expansion states.

## Highlights

• Medicaid enrollees post-Affordable Care Act had lower vaccination likelihoods.
• Medicaid enrollees in expansion states had lower vaccination likelihoods.
• Medicaid expansion per se did not increase pregnant women's vaccination likelihoods.
• Medicaid enrollees in expansion states need non-income-based immunization policies.
• Vulnerable pregnant women in non-expansion high-poverty states need interventions.

## 1 Introduction

With the looming Affordable Care Act (ACA) and Medicaid cuts in the United States (US), (Levitt, 2024) it is essential to examine whether Medicaid expansion, which increased the financial eligibility of low-income individuals, has affected US healthcare utilization for evidence-based policymaking. Medicaid, established in 1965 under Title XIX of the Social Security Act, provides federally and state-funded, state-administered health insurance for eligible low-income individuals, including children, pregnant women, parents, seniors, and individuals with disabilities (CDC, 2022). Eligibility is based on income and nonfinancial factors, including state residency, age, pregnancy, parenting, and immigration status (*Medicaid. Eligibility policy | Medicaid. Medicaid.gov. Published*, 2022). Initially, the average Federal Poverty Level (FPL) eligibility was below 50 % (Paradise et al., 2015). The ACA (*Patient protection and affordable care act. Govinfo.gov. Published*, 2010; *Coverage of Certain Preventive Services Under the Affordable Care Act. Federal Register. Published July* 14, 2015) amended Title XIX, effective in January 2014, for states to voluntarily expand Medicaid to adults (*Medicaid expansion to the new adult group. MACPAC. Published March* 23, 2023) with income below 138 % of the FPL (MACPAC, 2022). The federal government provides a higher matching rate of 90 % for adults in expanded Medicaid, including those who become pregnant (*Medicaid expansion to the new adult group. MACPAC. Published March* 23, 2023; Harker and Sharer, 2024). More relatively low-income pregnant women could gain eligibility and thus access to care that reduces health risks, such as vaccination. The Centers for Disease Control and Prevention (CDC) recommends influenza and tetanus–diphtheria–acellular pertussis (Tdap) vaccines for pregnant women to protect both mothers and infants (CDC, 2022; Hebballi et al., 2022; Ghosh et al., 2024). We examine the effects of Medicaid expansion on vaccination among pregnant women.

However, no peer-reviewed articles exist on such effects for pregnant women, except for eight studies on other populations to date, including teens and non-pregnant adult women (Tummalapalli and Keyhani, 2020; Song and Kucik, 2022; Han et al., 2015; Hatch et al., 2021; Agénor et al., 2023; Hawkins et al., 2021; Hoff et al., 2020; Churchill, 2021). Four studies examined Medicaid expansion impacts on influenza vaccination and found mixed results: Two found positive and significant effects using 2012–15 electronic health records data on 14 states and multivariate logistic regression (Han et al., 2015), and 2010–12 Medical Expenditure Panel Survey data on 50 states and the District of Columbia with a difference-in-difference estimation method (Hatch et al., 2021); One found negative and insignificant impacts using 2011–19 Behavioral Risk Factor Surveillance System (BRFSS) data on 33 states and difference-in-difference estimation (Song and Kucik, 2022); One found negative and insignificant impacts initially after the ACA implementation (2014–15) but positive and significant impact later (2016–17) using 2012–17 BRFSS data on 46 states and difference-in-difference estimation (Tummalapalli and Keyhani, 2020). Four studies examined the impacts of Medicaid expansion on Human Papillomavirus vaccination (Agénor et al., 2023; Hawkins et al., 2021; Hoff et al., 2020; Churchill, 2021). Five examined the racial and ethnic heterogeneous effects (Tummalapalli and Keyhani, 2020; Song and Kucik, 2022; Han et al., 2015; Hatch et al., 2021; Agénor et al., 2023). None included American Indians and Alaska Natives (AIANs) as a distinct racial category.

To fill the literature gap, we made four contributions. First, we added different national data on unstudied populations—pregnant women and AIANs—using the Pregnancy Risk Assessment Monitoring System (PRAMS). Second, we were the first to examine the effects of Medicaid expansion on Tdap vaccination of pregnant women. Third, we addressed mixed evidence on influenza vaccination. Fourth, we employed a triple-difference (DDD) estimation method, a quasi-experimental design for causal inference, in addition to the difference-in-difference method in the literature.

## 2 Materials and methods

### 2.1 Data and study population

We used 2011–15 PRAMS data on pregnant women in all 50 U.S. states and the District of Columbia, the only maternal and infant health surveillance nationwide jointly administered by state health departments and the CDC. PRAMS collects data monthly through stratified random sampling from current birth certificates of each jurisdiction, mail surveys, and follow-up telephone interviews. PRAMS is frequently used to evaluate state- and national-level health programs and policies (Shulman et al., 2018). State socioeconomic and demographic variables were extracted from the widely used longitudinal National Welfare Data aggregated from administrative data by the University of Kentucky Center for Poverty Research (UKCPR) (University of Kentucky Center for Poverty Research, 2024). Individual-level PRAMS and state-level UKCPR data were merged based on state residency, resulting in a final dataset of 44,320 pregnant women. The study period included three years before (2011−13) and two years after (2014–15) ACA implementation. It started in 2011 because PRAMS data introduced the influenza vaccination variable first in 2009 and to all states in 2011. The study ended in 2015 for unbiased estimates, as the federal recommendation changed against using live attenuated influenza vaccine in 2016 (Grohskopf et al., 2016b). Our health economic study is a secondary data analysis of existing, publicly available, de-identified data. Generally, secondary data analyses in economics do not require Institutional Review Board approval, especially when the data are publicly available and de-identified, as is the case in our study.

### 2.2 Study design

We employed a DDD estimation method, a quasi-experimental design for causal inference. The first difference is represented by the “Post ACA” time indicator variable, capturing the change in individual vaccination likelihood before and after the ACA implementation. An indicator variable takes a binary value (0,1). The second difference is represented by the “Expansion State” indicator variable, indicating the difference in individual vaccination likelihood between states that have and have not expanded Medicaid as of January 1, 2014. The third difference is the “Medicaid Insurance” indicator variable, measuring the difference in individual vaccination likelihood between Medicaid and non-Medicaid enrollees. Therefore, the Medicaid expansion treatment is a three-way interaction among time, state, and Medicaid insurance indicators. It takes value one for individuals who are 1) Medicaid enrollees, 2) in an expansion state, and 3) after the ACA implementation; it takes value zero otherwise. In other words, the Medicaid expansion treatment group consists of individuals who are Medicaid enrollees in expansion states after the ACA implementation. The control group consists of both non-Medicaid enrollees in expansion states and all individuals in non-expansion states.

### 2.3 Statistical analysis, outcome variables and covariates

(1) $${\mathrm{Vac}}_{\mathrm{ijt}}=\alpha +\beta_{1}\;\left[\mathrm{Post}\;\mathrm{ACA}\times \mathrm{Exp}\;\mathrm{State}\times \mathrm{Medicaid Insurance}\right]+\beta_{2}\;\left[\mathrm{Post}\;\mathrm{ACA}\times \mathrm{Exp}\;\mathrm{State}\right]+\beta_{3}\;\left[\mathrm{Post}\;\mathrm{ACA}\times \mathrm{Medicaid Insurance}\right]+\beta_{4}\;\left[\mathrm{Exp}\;\mathrm{State}\times \mathrm{Medicaid Insurance}\right]+\beta_{5}\;\left[\mathrm{Medicaid Insurance}\right]+\beta_{6}\;\left[\mathrm{Exp}\;\mathrm{State}\right]+\beta_{7}\;\left[\mathrm{Post}\;\mathrm{ACA}\right]+X_{\mathrm{it}}\theta +\delta_{s}+\tau_{t}+\varepsilon_{\mathrm{ijt}.}$$

We used the specification Eq. (1) and estimated it using the linear probability model. The main outcome variable, *Vac*_*ijt*_, is a pregnant woman *i*'s vaccination status in year *t* for vaccine *j* = {Tdap, Influenza}. The coefficient *β*_*1*_ of the three-way interaction term measures the treatment effect of Medicaid expansion on the individual vaccination probability of a pregnant woman. That is, it measures the effect of Medicaid expansion on the individual vaccination probability of a pregnant woman in the treatment group, compared to a pregnant woman in the control group.

Then we add three two-way interactions. The coefficient *β*_*2*_ captures the impact of the ACA implementation and residency in an expansion state on the individual vaccination probability. That is, it captures the individual vaccination probability of a pregnant woman residing in an expansion state after the ACA implementation, compared to a pregnant woman residing in a non-expansion state or before the implementation. Similarly, the coefficient *β*_*3*_ represents the impact of the ACA implementation and Medicaid enrollment on the probability. That is, it represents the individual vaccination probability of a pregnant woman enrolled in Medicaid after the ACA implementation, compared to a pregnant woman not enrolled in Medicaid or before the implementation. The coefficient *β*_*4*_ measures the impact of residency in an expansion state and Medicaid enrollment on the probability. That is, it measures the individual vaccination probability of a pregnant woman enrolled in Medicaid in an expansion state, compared to a pregnant woman not enrolled in Medicaid or in a non-expansion state.

Then we add three single indicator variables. The coefficients *β*_*5*_, *β*_*6*_, and *β*_*7*_ capture the impacts of Medicaid enrollment, residency in an expansion state, and the ACA implementation on the individual vaccination probability, respectively. The latter two indicators were captured by the state- and year-fixed effects. Estimates of the above four interaction terms and the Medicaid indicator are crucial to answering our research question and will be reported in Table 2, Table 3.

In addition, we included individual indicators: *X*_*it*_ denotes a set of state- and individual-level control variables, including proportion of state residents with incomes below the federal poverty level, state minimum wage, state population, the number of state Supplemental Nutrition Assistance Program recipients, as well as racial indicators (AIAN as a distinct category), a parity indicator, and a college education indicator. Including state- and individual-level indicators can show additional interesting results for policy implications. Estimates of these additional indicators are reported in full versions of Table 2, Table 3 in Appendix Tables A2 and A3, where the first column reports estimates of state-level indicators, and the second column reports estimates of individual-level indicators. Consistency of estimates of the four interaction terms across these columns adjusted for state- and individual-level indicators also serves as the sensitivity tests.

Finally, *δ*_*s*_ denotes state-fixed effects, addressing any time-invariant, state-specific, unobserved heterogeneity. τ_*t*_ accounts for any shocks affecting all states in a given year of the study period. Standard errors are clustered at the state level. STATA 18.5 was used for statistical analysis.

## 3 Results

Table 1 summarizes the data, including 10,594 participants in the treatment group and 33,726 participants in the control group. It shows mean differences between the two groups. Compared to the control group, the treatment group exhibited 0.24 (*P* < 0.01) and 0.13 (P < 0.01) higher proportions of pregnant women who received Tdap and influenza vaccination during pregnancy, respectively, which suggests potential treatment effects of Medicaid expansion and motivates the following empirical estimations.

**Table 1:** Summary statistics: Proportions of pregnant women who received Tetanus, Diphtheria and Acellular Pertussis and Influenza vaccination and socioeconomic and demographic characteristics of United States pregnant women by Medicaid expansion treatment and control groups, 2011–2015.

	Control Group Mean	Treatment Group Mean	Difference in Means
	(SD)	(SD)	(*P*-value)
Proportion of pregnant women who received Tdap vaccination during pregnancy	0.29	0.52	0.24
	(0.45)	(0.50)	(*<*0.01)
Proportion of pregnant women who received influenza vaccination during pregnancy	0.34	0.47	0.13
	(0.47)	(0.49)	(*<*0.01)
Proportion of State Residents with Medicaid Insurance Coverage	0.23	0.27	0.04
	(0.42)	(0.44)	(*<*0.01)
Proportion of African American Individuals	0.17	0.12	−0.05
	(0.37)	(0.33)	(*<*0.01)
Proportion of White Individuals	0.66	0.74	0.08
	(0.47)	(0.44)	(*<*0.01)
Proportion of American Indian and Alaska Native Individuals (AIAN)	0.03	0.02	−0.005
	(0.16)	(0.14)	(0.001)
Proportion of Asian Individuals	0.04	0.04	0.003
	(0.19)	(0.20)	(0.24)
Proportion of Individuals with Child Less Than 2 Years	0.73	0.74	0.01
	(0.45)	(0.44)	(0.15)
Proportion of Individuals with College Degrees and Above	0.33	0.38	0.05
	(0.46)	(0.48)	(*<*0.01)
Log State Population	15.35	15.44	0.09
	(0.81)	(1.16)	(*<*0.01)
State Minimum Wage ($)	7.37	8.03	0.65
	(0.44)	(0.39)	(*<*0.01)
Proportion of state residents with incomes below the federal poverty level	13.21	12.78	−0.43
	(2.48)	(2.88)	(*<*0.01)
Proportion of state residents enrolled in SNAP	13.41	13.55	0.14
	(0.80)	(1.13)	(*<*0.01)
Number of Observations	33,726	10,594	

Sources: Individual-level vaccination, insurance, and control variables are obtained from 2011 to 2015 CDC-PRAMS data. State-level controls are obtained from the UKCPR National Welfare data. Data are merged by state residency of each individual. Notes: The numbers in the parentheses of the third column denote *P*-values of the *t*-test of differences in mean values of control and treatment groups of individual pregnant women. The Medicaid expansion treatment group consists of individuals who are Medicaid enrollees in expansion states after the ACA implementation. The control group consists of both non-Medicaid enrollees in expansion states and all individuals in non-expansion states. Tdap: Tetanus, Diphtheria and Acellular Pertussis; SNAP: Supplemental Nutrition Assistance Program; CDC-PRAMS: Centers of Disease Control and Prevention - Pregnancy Risk Assessment Monitoring system; UKPCR: University of Kentucky Center for Poverty Research.

We report and interpret the coefficients of our DDD estimation method here and then provide economic explanations in the discussion section. We first report DDD estimates of the Medicaid expansion treatment effect in row 1 in the following Tables. In Table 2, the coefficient was statistically insignificant at −0.03. This indicates that the Medicaid expansion did not significantly affect the likelihood of a pregnant woman receiving the Tdap vaccination, possibly because non-Medicaid enrollees had a relatively high vaccination propensity, which is consistent with the *β*_*5*_ estimate reported and discussed below. Similarly, Table 3 reports the DDD estimates for influenza vaccination. The coefficient was statistically insignificant at 0.02. Therefore, the Medicaid expansion did not significantly impact both vaccination likelihoods of a pregnant woman.

**Table 2:** Triple-difference linear probability estimates of the Medicaid expansion treatment effect on Tetanus, Diphtheria and Acellular Pertussis Vaccination of an individual United States pregnant woman, by state- and individual-level characteristics, with state and time fixed effects, 2011–2015.

	Dependent Variable: Tdap Vaccination
	(1)
	−0.03
Three-way interaction term (enrolled in Medicaid * residence in an expansion state * post expansion year)	(0.02)
	0.019
Two-way interaction term (residence in an expansion state * post expansion year)	(0.04)
	−0.09^⁎⁎⁎^
Two-way interaction term (post expansion year * enrolled in Medicaid)	(0.02)
	0.01
Two-way interaction term (residence in an expansion state * enrolled in Medicaid)	(0.02)
Medicaid insurance indicator	0.01
	(0.02)
	
Observations	44,012
F Statistic (p-value)	182.74 (<0.01)
State Fixed Effects	Y
Year Fixed Effects	Y

Sources: Individual-level vaccination, insurance, and control variables are obtained from 2011 to 2015 CDC-PRAMS data. State-level controls are obtained from the UKCPR National Welfare data. Data are merged by state residency of each individual. Notes: Table 2 reports estimates whether Medicaid expansion significantly affected the Tdap vaccination likelihood of a pregnant woman. Standard errors in parentheses. * *p* < 0.10, ** *p* < 0.05, *** *p* < 0.01. All specifications include state and year fixed effects. Standard errors are clustered at the state level. ACA: Affordable Care Act; SNAP: Supplemental Nutrition Assistance Program; FE: Fixed effects; CDC-PRAMS: Centers of Disease Control and Prevention - Pregnancy Risk Assessment Monitoring system; UKPCR: University of Kentucky Center for Poverty Research. An indicator is coded either 0 or 1. The model is adjusted for individual indicators. The full version of this table in Appendix Table A2 reports estimates for all individual indicators.

**Table 3:** Triple-difference linear probability estimates of the Medicaid expansion treatment effect on influenza vaccination of an individual United States pregnant woman, by state- and individual-level characteristics, with state and time fixed effects, 2011–2015.

	Dependent Variable: Flu Vaccination
	(1)
	0.02
Three-way interaction term (enrolled in Medicaid * residence in an expansion state * post expansion year)	(0.02)
	−0.02
Two-way interaction term (residence in an expansion state * post expansion year)	(0.01)
	−0.04^⁎⁎^
Two-way interaction term (post expansion year * enrolled in Medicaid)	(0.02)
	−0.03^⁎^
Two-way interaction term (residence in an expansion state * enrolled in Medicaid)	(0.01)
Medicaid insurance indicator	0.001
	(0.01)
	
Observations F Statistic (p-value)	44,012 151.21 (<0.01)
State Fixed Effects	Y
Year Fixed Effects	Y

Sources: Individual-level vaccination, insurance, and control variables are obtained from 2011 to 2015 CDC-PRAMS data. State-level controls are obtained from the UKCPR National Welfare data. Data are merged by state residency of each individual. Notes: Table 3 reports estimates whether Medicaid expansion significantly affected the influenza vaccination likelihood of a pregnant woman. Standard errors in parentheses. * p < 0.10, ** p < 0.05, *** p < 0.01. All specifications include state and year fixed effects. Standard errors are clustered at the state level. ACA: Affordable Care Act; SNAP: Supplemental Nutrition Assistance Program; FE: Fixed effects; CDC-PRAMS: Centers of Disease Control and Prevention - Pregnancy Risk Assessment Monitoring system; UKPCR: University of Kentucky Center for Poverty Research. An indicator is coded either 0 or 1. The model is adjusted for individual indicators. The full version of this table in Appendix Table A3 reports estimates for all individual indicators.

We then report *β*_*2*_, *β*_*3*_, and *β*_*4*_ estimates in rows 2–4. In Table 2, row 2, the *β*_*2*_ estimate was not statistically significant at the 0.02 level, suggesting no measurable difference. Similarly, in Table 3, its estimate had no significance after incorporating fixed effects and controls. Thus, no statistical differences existed in both vaccination likelihoods between a pregnant woman in an expansion state after the ACA and a counterpart in a non-expansion state before ACA implementation.

In Table 2, row 3, the *β*_*3*_ estimate was stably near −0.09 (*P* < 0.01). This indicates that a Medicaid-enrolled pregnant woman after the ACA was approximately 9.00 % less likely to get Tdap vaccination than a non-Medicaid enrollee before ACA implementation. Similarly, Table 3 suggests that a Medicaid-enrolled pregnant woman after the ACA was 4.00 % (*P* < 0.05) less likely to receive an influenza vaccination than a non-Medicaid enrollee before the ACA.

In Table 2, row 4, the *β*_*4*_ estimates were statistically insignificant at 0.01. Throughout the study period, no differences existed in Tdap vaccination likelihood between a pregnant woman Medicaid enrolled in an expansion state and non-Medicaid enrolled in a non-expansion state. However, in Table 3, the coefficient was −0.03 (*P* < 0.1), suggesting that a Medicaid-enrolled pregnant woman in an expansion state is 3.00 % less likely to take influenza vaccination than a non-Medicaid enrollee in a non-expansion state.

For the Medicaid insurance indicator in Table 2, the *β*_*5*_ estimate suggests no measurable difference in the likelihood of Tdap vaccination between Medicaid- and non-Medicaid-enrolled pregnant women. Similar findings were reported in Table 3.

Coefficient estimates of the expansion state and post-ACA time indicators were automatically dropped in Table 2, Table 3 due to the incorporation of state and year fixed effects. The inclusion of state and year-fixed effects was strongly supported by the F-tests (*P* < 0.01). Row 6 shows that a pregnant woman in an expansion state was 7.30 % and 14.20 % (*P* < 0.05) more likely than a counterpart in a non-expansion state to receive Tdap and influenza vaccinations. Row 7 suggests that a pregnant woman after the ACA was 30.30 % and 19.20 % (*P* < 0.01) more likely than a counterpart before the ACA to receive Tdap and influenza vaccinations.

The full versions of Table 2, Table 3 show interesting results of additional individual indicators (see Appendix). Coefficient estimates for the African American indicator ranged between −0.04 and − 0.05 (P < 0.01) for both vaccinations. In contrast, those of the AIAN indicator were at 0.04 (P < 0.05) and 0.07 (P < 0.01) for Tdap and influenza vaccinations. The college education indicator was estimated at 0.05 and 0.11 (P < 0.01) for Tdap and influenza vaccinations, respectively. The first or second pregnancy indicator was at 0.05 and 0.03 (P < 0.01) for Tdap and influenza vaccinations. The proportion of state residents with incomes below the federal poverty level was estimated at −0.01 (P < 0.01) for influenza vaccination but was insignificant for Tdap vaccination.

## 4 Discussion

Findings of the three single indicator variables are intuitive, including ACA implementation, residency in an expansion state, and Medicaid enrollment. A pregnant woman in the post-ACA period or an expansion state was more likely to vaccinate against both influenza and COVID-19 than a counterpart in the pre-ACA period or a non-expansion state, which is consistent with the literature indicating that a parent in an expansion state was 6.7 % more likely to vaccinate against influenza (Glied and Weiss, 2023). Although Medicaid-insured pregnant women had lower population vaccination coverage than those with private insurance (Ghaswalla et al., 2019; Moll et al., 2021), our findings suggest individual vaccination probabilities of Medicaid and non-Medicaid enrollees may not differ significantly.

The findings of the two- and three-way interactions are consistent with the limited existing literature and call for future research on the mechanisms involved. Firstly, there were no differences in vaccinations between pregnant women in expansion states after the ACA and those in non-expansion states before the ACA. Although expansion states may have more healthcare infrastructure (Harker and Sharer, 2024) and access to care, they may also have more low-income, Medicaid-eligible and enrolled individuals, whose vaccination differs from those of high-income individuals, with potential mechanisms below.

Secondly, Medicaid-enrolled pregnant women after the ACA were less likely to vaccinate than non-Medicaid enrollees before the ACA, possibly because post-ACA Medicaid enrollees were relatively low-income compared to those already enrolled in non-Medicaid (mostly private in PRAMS) insurance before the ACA. Income may affect vaccination through three mechanisms: 1) the slope and 2) the acceleration of the income-vaccination relationship, given vaccination as consumption; 3) vaccination as an investment (Zhao et al., 2018). First, low-income women had vaccine hesitancy, thus leading to vaccination delay and non-adherence in consumption behavior (Gilbert et al., 2021). Second, the income elasticity of demand for vaccination measures how much demand for vaccination changes with income. We hypothesize that if the income of a low-income individual decreases by one unit, their demand for vaccination decreases by more than one unit (Sauerborn et al., 1994). Third, according to the health capital theory, lower-wage individuals invest less in health than higher-wage individuals because the earning return on investment is lower (Grossman, 1972). We hypothesize that low-income individuals face tight budget constraints and prioritize the consumption of basic goods over investments like vaccination, which reduces future health and associated financial losses while generating long-term health and productivity gains and associated earnings returns (Zhao et al., 2024). Future empirical research is needed to test both hypotheses.

Thirdly, Medicaid-enrolled pregnant women in expansion states were less likely than non-Medicaid enrollees in non-expansion states to take influenza vaccination, with no difference in Tdap vaccination. This suggests that the positive effect of the state indicator above depends on whether an individual is Medicaid enrolled in Medicaid. Individuals newly eligible and enrolled in Medicaid in expansion states are relatively low-income compared to non-Medicaid (mostly private in PRAMS) insurance enrollees in non-expansion states, thus again revealing low vaccination behavior, which is possible through the income mechanisms elaborated above. This low vaccination likelihood only applied to influenza, rather than Tdap, possibly because pregnant women perceived pertussis as a serious condition and influenza vaccines as unsafe, according to a randomized controlled trial (Chamberlain et al., 2015). Lastly, Medicaid expansion had no treatment effects on both vaccinations of pregnant women, possibly because non-Medicaid enrollees had a relatively high vaccination propensity compared to Medicaid enrollees. This finding is consistent with our results for the Medicaid indicator and the income mechanisms discussed above, and it provides new empirical evidence to address the missing and mixed evidence in the literature.

Associations varied across individual- and state-level demographic (e.g., racial) and socioeconomic characteristics. African American pregnant women had a lower likelihood to vaccinate than non-African American women, possibly due to access barriers, such as living in medically underserved areas and having historical mistrust in healthcare (Brown et al., 2016; Bajaj and Stanford, 2021). AIAN pregnant women had a higher likelihood to vaccinate than non-AIAN women, possibly because of Medicaid's AIAN exemption from out-of-pocket costs (*Out-of-Pocket Cost Exemptions | Medicaid*, 2025) and successful tribal and federal health interventions (Jarshaw et al., 2024). However, the AIAN population still has lower overall vaccine coverage than the general US population (Flu Vaccination Coverage, n.d.). Individuals with a college education had a higher likelihood of vaccination, possibly because people with limited health literacy have a reduced capacity to understand the benefits and risks of vaccines (Le et al., 2020). Those with low parity were more likely to vaccinate than those with high parity, possibly because the latter pay less attention to prevention during the current pregnancy or have experienced no infections (Descamps et al., 2019) or received vaccinations during prior pregnancies and thus have no perceived needs (Strassberg et al., 2018). Those in high-poverty states were less likely than those in low-poverty states to receive influenza vaccination, with no difference in Tdap vaccination. This may be due to the lower healthcare infrastructure, and reducedin poorer states (Hsia et al., 2011) and more severity perception of the Tdap condition and safety concerns about the influenza vaccine (Hsia et al., 2011).

Clinical and policy implications of findings suggest a need for non-income-based policies targeting Medicaid-enrolled pregnant women in expansion states for preventive services. For example, interventions may educate these women on preventive care as a means of investing in their health capital. Such interventions may focus on influenza vaccination through consumer hesitancy surveys and physician recommendations (Influenza, 2023) and standing orders. Future policies may also target pregnant women in non-expansion states, regardless of insurance type, who were less likely to vaccinate than those in expansion states, in general. Future interventions may also target pregnant women who are African Americans, with low education, with high parity, or in high-poverty states. Future empirical research should test both hypotheses regarding elastic demand for vaccination and the lack of understanding of vaccination as a health investment among low-income individuals.

Limitations exist. PRAMS data are weighted by jurisdiction, while their aggregate data are not weighted to be fully nationally representative. However, PRAMS data represent about 83 % of all live births in the US, thus highly representative of most national analyses related to pregnancy and childbirth (Shulman et al., 2018). The strengths of PRAMS data also include the large sample size of pregnant women and detailed information on vaccination status, insurance types, and maternal and infant health outcomes; these are less available in BRFSS data and more suitable for our study population. (Federal Register: Request Access, 2023; Stoecker et al., 2017).

## 5 Conclusions

Using different national data on unstudied populations (pregnant women and AIANs) and the triple-difference estimation method, we added new empirical evidence to address missing and mixed evidence in the literature. Medicaid expansion did not increase the likelihoods of pregnant women taking Tdap and influenza vaccinations recommended by the CDC. Non-income-based policies may target Medicaid enrollees in expansion states, especially for influenza vaccination. Findings also identify pregnant women in non-expansion states, regardless of insurance type, with high state poverty, high parity, low education, or who are African Americans, for future interventions.

## CRediT authorship contribution statement

**Junying Zhao:** Writing – review & editing, Writing – original draft, Validation, Supervision, Resources, Methodology, Investigation, Funding acquisition. **Rashmi Jaggad:** Writing – review & editing, Writing – original draft, Methodology, Data curation, Conceptualization. **Ahmed El Fatmaoui:** Visualization, Validation, Software, Methodology, Investigation, Formal analysis, Data curation. **Pallab K. Ghosh:** Writing – review & editing, Supervision, Software, Methodology, Investigation, Formal analysis, Data curation.

## Declaration of competing interest

The authors declare that they have no known competing financial interests or personal relationships that could have appeared to influence the work reported in this paper.

## Data Availability

Data will be made available on request.

## Appendix A Supplementary data

Supplementary material Image 1
