# Supplementary material for: Did the Medicaid expansion improve immunization among U.S. pregnant women?
Source: Prev Med Rep. 2025 Aug 20;58:103214. doi: 10.1016/j.pmedr.2025.103214 (PMC12491732; doi:10.1016/j.pmedr.2025.103214)
Supplement: Supplementary file 1 — Supplementary material [file mmc1.docx]

**Appendix**

Table A2: Triple-difference linear probability estimates of the Medicaid expansion treatment effect on Tetanus, Diphtheria and Acellular Pertussis Vaccination of an individual United Statespregnant woman, by state- and individual-level characteristics, with state and time fixed effects, 2011-2015.

|  | Dependent Variable: Tdap Vaccination | | |
| --- | --- | --- | --- |
|  | (1) | (2) | (3) |
|  | -0.03 | -0.03 | -0.03 |
| Three-way interaction term (enrolled in Medicaid * residence in an expansion state * post expansion year) | (0.02) | (0.02) | (0.02) |
|  | 0.02 | 0.02 | 0.019 |
| Two-way interaction term (residence in an expansion state * post expansion year) | (0.04) | (0.04) | (0.04) |
|  | -0.09^***^ | -0.09^***^ | -0.09^***^ |
| Two-way interaction term (post expansion year * enrolled in Medicaid) | (0.01) | (0.02) | (0.02) |
|  | 0.01 | 0.002 | 0.01 |
| Two-way interaction term (residence in an expansion state * enrolled in Medicaid) | (0.02) | (0.02) | (0.02) |
| Medicaid insurance indicator | -0.02 | -0.01 | 0.01 |
|  | (0.02) | (0.02) | (0.02) |
| Log state population | -1.09 | -1.10 | -1.15 |
|  | (1.47) | (1.47) | (1.45) |
| State minimum wage | 0.01 | 0.01 | 0.01 |
|  | (0.04) | (0.04) | (0.04) |
|  | -0.01 | -0.01 | -0.01 |
| Proportion of state residents with incomes below the federal poverty level | (0.00) | (0.01) | (0.01) |
| Log number of state SNAP recipients | -0.04 | -0.04 | -0.04 |
|  | (0.13) | (0.13) | (0.12) |
| African American race indicator |  | -0.05^***^ | -0.05^***^ |
|  |  | (0.01) | (0.01) |
| White race indicator |  | -0.01 | -0.02 |
|  |  | (0.02) | (0.02) |
| American Indians and Alaska Natives race indicator |  | 0.04^**^ | 0.03^**^ |
|  |  | (0.02) | (0.02) |
| Asian American race indicator |  | -0.01 | -0.04^*^ |
|  |  | (0.02) | (0.02) |
| First or second pregnancy indicator |  |  | 0.05^***^ |
|  |  |  | (0.01) |
| College education indicator |  |  | 0.05^***^ |
|  |  |  | (0.01) |
| Constant | 16.78 | 17.00 | 17.80 |
|  | (22.16) | (22.18) | (21.94) |
| Observations | 44,012 | 44,012 | 44,012 |
| F Statistic (p-value) | 203.82 (<0.01) | 184.85 (<0.01) | 182.74 (<0.01) |
| State Fixed Effects | Y | Y | Y |
| Year Fixed Effects | Y | Y | Y |

Sources: Individual-level vaccination, insurance, and control variables are obtained from 2011-2015 CDC-PRAMS data. State-level controls are obtained from the UKCPR National Welfare data. Data are merged by state residency of each individual. Notes: Table 2 reports estimates whether Medicaid expansion significantly affected the Tdap vaccination likelihood of a pregnant woman. Standard errors in parentheses. * p<0.10, ** p<0.05, *** p<0.01. All specifications include state and year fixed effects. Standard errors are clustered at the state level. ACA: Affordable Care Act; SNAP: Supplemental Nutrition Assistance Program; FE: Fixed effects; CDC-PRAMS: Centers of Disease Control and Prevention - Pregnancy Risk Assessment Monitoring system; UKPCR: University of Kentucky Center for Poverty Research. An indicator is coded either 0 or 1.

Table A3: Triple-difference linear probability estimates of the Medicaid expansion treatment effect on influenza vaccination of an individual United States pregnant woman, by state- and individual-level characteristics, with state and time fixed effects, 2011-2015.

|  | Dependent Variable: Flu Vaccination | | |
| --- | --- | --- | --- |
|  | (1) | (2) | (3) |
|  | 0.02 | 0.02 | 0.02 |
| Three-way interaction term (enrolled in Medicaid * residence in an expansion state * post expansion year) | (0.02) | (0.02) | (0.02) |
|  | -0.02 | -0.02 | -0.02 |
| Two-way interaction term (residence in an expansion state * post expansion year) | (0.01) | (0.01) | (0.01) |
|  | -0.04^**^ | -0.04^**^ | -0.04^**^ |
| Two-way interaction term (post expansion year * enrolled in Medicaid) | (0.02) | (0.02) | (0.02) |
|  | -0.03^*^ | -0.03^**^ | -0.03^*^ |
| Two-way interaction term (residence in an expansion state * enrolled in Medicaid) | (0.02) | (0.01) | (0.01) |
| Medicaid insurance indicator | -0.05^***^ | -0.03^***^ | 0.001 |
|  | (0.01) | (0.01) | (0.01) |
| Log state population | -0.06 | -0.07 | -0.16 |
|  | (0.50) | (0.50) | (0.50) |
| State minimum wage | 0.04^*^ | 0.03 | 0.03 |
|  | (0.02) | (0.02) | (0.02) |
|  | -0.01^***^ | -0.01^***^ | -0.01^***^ |
| Proportion of state residents with incomes below the federal poverty level | (0.00) | (0.00) | (0.00) |
| Log number of state SNAP recipients | 0.101 | 0.10 | 0.01 |
|  | (0.17) | (0.17) | (0.17) |
| American Indians and Alaska Natives race indicator |  | -0.04^***^ | -0.04^***^ |
|  |  | (0.01) | (0.01) |
| White race indicator |  | 0.03^***^ | 0.01 |
|  |  | (0.01) | (0.01) |
| American Indian race indicator |  | 0.07^***^ | 0.07^***^ |
|  |  | (0.02) | (0.02) |
| Asian American race indicator |  | 0.06^***^ | 0.02 |
|  |  | (0.02) | (0.02) |
| First or second pregnancy indicator |  |  | 0.03^***^ |
|  |  |  | (0.01) |
| College education indicator |  |  | 0.11^***^ |
|  |  |  | (0.01) |
| Constant | -0.34 | -0.23 | 1.09 |
|  | (6.33) | (6.33) | (6.15) |
| Observations  F Statistic (p-value) | 44,012  155.59 (<0.01) | 44,012  144.10 (<0.01) | 44,012  151.21 (<0.01) |
| State Fixed Effects | Y | Y | Y |
| Year Fixed Effects | Y | Y | Y |

Sources: Individual-level vaccination, insurance, and control variables are obtained from 2011-2015 CDC-PRAMS data. State-level controls are obtained from the UKCPR National Welfare data. Data are merged by state residency of each individual. Notes: Table 3 reports estimates whether Medicaid expansion significantly affected the influenza vaccination likelihood of a pregnant woman. Standard errors in parentheses. * p<0.10, ** p<0.05, *** p<0.01. All specifications include state and year fixed effects. Standard errors are clustered at the state level. ACA: Affordable Care Act; SNAP: Supplemental Nutrition Assistance Program; FE: Fixed effects; CDC-PRAMS: Centers of Disease Control and Prevention - Pregnancy Risk Assessment Monitoring system; UKPCR: University of Kentucky Center for Poverty Research. An indicator is coded either 0 or 1.
